# Effects of preoperative oral management by dentists on postoperative outcomes following esophagectomy: Multilevel propensity score matching and weighting analyses using the Japanese inpatient database: Erratum

**DOI:** 10.1097/MD.0000000000017243

**Published:** 2019-09-13

In the article “Effects of preoperative oral management by dentists on postoperative outcomes following esophagectomy: Multilevel propensity score matching and weighting analyses using the Japanese inpatient database”,^[1]^ which appears in Volume 98, Issue 17 of *Medicine*, there was an error in Abstract. The first sentence of the fourth paragraph of the Abstract should appear as “We analyzed 2600 esophagectomy cases of which 956 were open, and 1644 were thoracoscopic surgery.” The authors apologize for this error and state that this does not change the scientific conclusions of the article in any way.
